# Displacement of the retina in the late follow-up after retinal detachment repair

**DOI:** 10.1007/s00417-025-06892-9

**Published:** 2025-09-15

**Authors:** Vilde M. Thomseth, Eduardo Roditi, Koby Brosh, Michael J. Potter, Vegard A. Forsaa

**Affiliations:** 1 Department of Ophthalmology, Stavanger University Hospital, Stavanger, Norway; 2 Department of Quality and Health Technology, University of Stavanger, Stavanger, Norway; 3 Department of Clinical Medicine, Section of Ophthalmology, University of Bergen, Bergen, Norway; 4 Faculty of Health Sciences, Universidad Anahuac Mexico Norte, Mexico City, México; 5 Active Vision Center, Mexico City, México; 6 Department of Ophthalmology, Shaare Zedek Medical Center, Faculty of Medicine, Hebrew University of Jerusalem, Jerusalem, Israel; 7 Department of Ophthalmology and Visual Sciences, University of British Columbia, Vancouver, Canada; 8 Helse Stavanger HST, Torgveien 25, 4016 Stavanger, Norway

**Keywords:** Retinal displacement, IR homography, Infrared scanning laser ophthalmoscopy, Perfluocarbon liquid, Reghmatogenous retinal detachment, Late follow-up retinal displacement

## Abstract

**Purpose:**

To investigate a potential displacement of the retina in the late follow-up after retinal detachment repair using infrared (IR) homography.

**Methods:**

Prospective observational study with a two-year follow-up of patients with primary fovea-off reghmatogenous retinal detachment (RRD). Patients who developed macular structural disease during follow-up, including epiretinal membrane, and patients with insufficient image quality were excluded. Patients were imaged with IR scanning laser ophthalmology images at 1 and 24 months after pars plana vitrectomy and sulphur hexafluoride tamponade for RRD. Using custom software, the two IR images from the separate follow-up visits were superimposed to generate IR homography images. Proper alignment was ensured using choroidal and optic nerve landmarks. The main outcome measure was misalignment of the major retinal vessel arcades on IR homography.

**Results:**

36 patients completed the follow-up. 13 patients with a median age of 62 years (IQR = 11) were included. Retinal displacement between the 1st and 24th postoperative month was found in 8 patients (62%). Inter-grader agreement was κ = 0.831 (SE: 0.16), (*p* = 0.002). The direction of displacement occurred inferiorly in *n* = 5 cases (63%) and superiorly in *n* = 3 cases (38%). Use of perfluocarbon liquid was positively associated with late displacement (ϕ = 0.675, (*p* = 0.015).

**Conclusions:**

This proof-of-concept study found retinal displacement in the late follow-up after retinal detachment repair using a validated method designated IR homography. Use of perfluocarbon liquid may have a long-lasting effect on the plasticity of the retina, warranting future investigation.

## Key messages

*****What is known***:**

- It is well known that the retina may displace from its original position on the retinal pigment epithelium during and immediately after surgery for retinal detachment, but it has not yet been established whether the retina continues to displace in the late postoperative period.

*****What is new***:**

- By superimposing infrared images from the 1^st^ and 24^th^ months after retinal reattachment surgery and excluding patients who developed structural macular disease, we found a displacement of the retina in 62% of patients, and late displacement was positively associated with the use of perfluocarbon liquid.
- Late displacement of the retina after retinal detachment surgery is a new finding that helps elucidate the pathophysiology of retinal detachment, and use of perfluocarbon liquid may have a long-lasting effect on retinal plasticity.

## Introduction

In rhegmatogenous retinal detachment (RRD), tractional forces cause the neurosensory retina to dissect from the retinal pigment epithelium (RPE), and due to the retina’s viscoelastic properties, it has an appreciable capacity to stretch before breaking [1]. A retinal displacement from the pre-detachment position on the pigment epithelium after RRD was first detected by Shiragami et al. who found hyperfluorescent “retinal vessel printings” (RVPs) on fundus autofluorescence (FAF) images in place of the original positions of the retinal vessels [2, 3].

During retinal reattachment surgery, applied tractional forces may induce retinal displacement through the use of perfluorocarbon liquids (PFCL) and/or intraocular gas tamponades, which displace the subretinal fluid (SRF) away from their contact area, stretching the retina in the process [4–6]. After reattachment is achieved, gravity is hypothesized to contribute to additional stretch of the retinal tissue by pulling residual SRF downward [7–9].

Little information exists on the long-term displacement behavior of the retina in patients after RRD [10, 11]. Shiragami et al. noted a narrowing of the space between the RVPs and the retinal vessels in the period between 3 and 12 months after reattachment surgery, suggestive of a displacement of the retina in the direction toward its original position, warranting further investigation.

Recent evidence indicates that the use of FAF images to evaluate a change in retinal position by evaluating the presence of RPVs underestimates the true rate and extension of retinal displacement [12]. A novel, validated method can identify retinal displacement by combining two superimposed infrared (IR) scanning laser ophthalmoscopy (SLO) images, and has achieved a sensitivity and specificity of 100% and 98%, respectively [13]. Using this technique that we have called IR homography, the present study aimed to investigate the potential displacement of the retina between the 1st and 24th postoperative months after retinal detachment repair.

## Methods

Our study material was derived from a prospective observational study of patients with fovea-off RRD who presented to the vitreoretinal surgery center at the Department of Ophthalmology at Stavanger University Hospital, Norway, between June 2017 and December 2020 (NCT03187613, ClinicalTrials.gov). All participants gave informed consent prior to enrollment and the study adhered to the Declaration of Helsinki. The Committee for Medical and Health Research Ethics, Norway, approved the study (2017/730).

Patients underwent either pars plana vitrectomy (PPV) and intraocular gas tamponade (sulfur hexafluoride 26%) (SF6), PPV with silicone oil tamponade, or scleral buckle with retinal cryopexy, as per a previous publication [14]. Combined phacoemulsification and PPV was performed in patients with cataract.

All patients were imaged with swept source optical coherence tomography (SS-OCT) before reattachment surgery. Postoperatively at one-, three-, six-, 12- and 24-months, patients were imaged with cross-sectional OCT scans (Topcon DRI OCT Triton; Topcon Corp., Tokyo, Japan) and thirty-degree field of view IR SLO images (Heidelberg Spectralis HRA, Heidelberg Engineering GmbH, Heidelberg, Germany).

Inclusion criteria were a minimum age of 18 years and primary fovea-off RRD. Exclusion criteria were the development of potentially confounding macular structural disease during follow-up, including epiretinal membranes (ERMs), and insufficient image quality.

Pre- and perioperative assessments were age, sex, lens status, patient reported fovea-off duration, detachment extent in clock hours, location of retinal break(s) in clockhours, use of PFCL and proliferative vitreoretinopathy (PVR) grade 0–C. Postoperative assessments were displacement of the retina on IR homography composed from IR images captured at the 1-, and 24-month postoperative visits. Displacement of the retina was defined as misalignment of the major vessel arcades on IR homography.

### IR homography

**Fig. 1:**
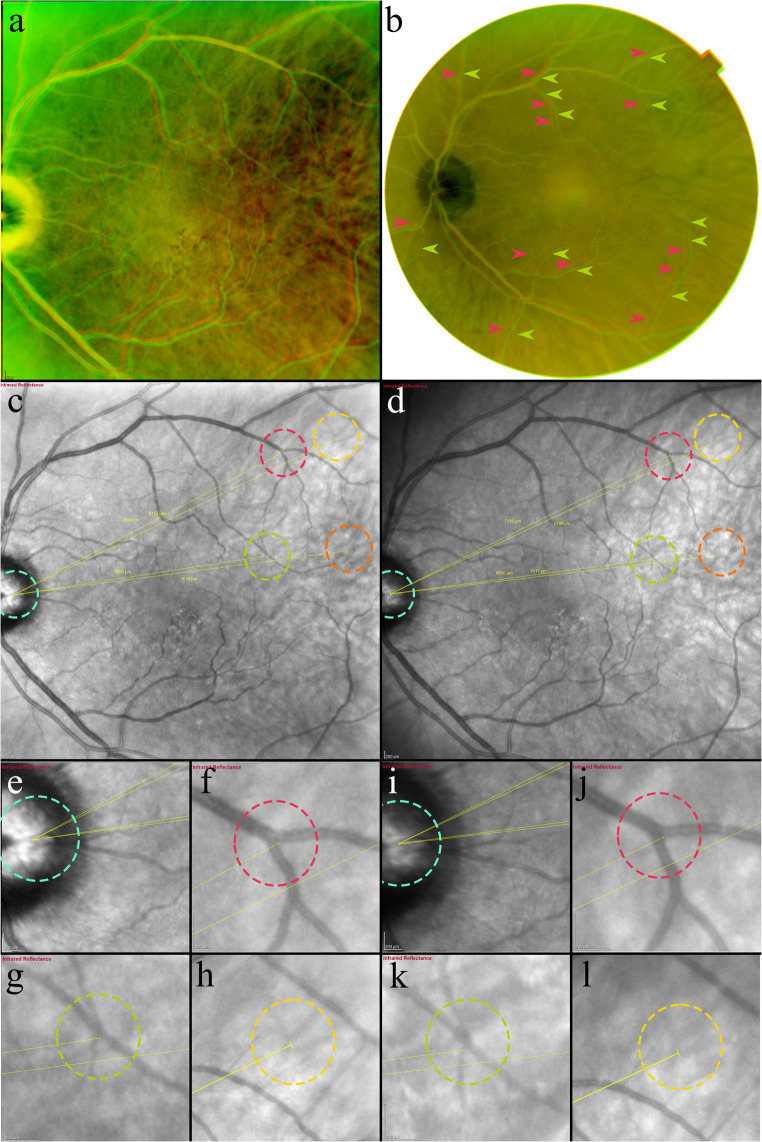
Multimodal homography demonstrating an inferotemporal retinal displacement in the late follow-up after retinal detachment repair. (**a**) Infrared homography composed of IR images from postoperative months 1 (red vessels) and 12 (green vessels). The temporal displacement direction is visible as misalignment of the vertically directed branches off the superior and inferior major vessel arcades. (**b**) Color fundus photography homography composed from color fundus photography images from postoperative months 1 (red vessels marked with red arrows) and 12 (green vessels marked with green arrows), demonstrating consistency in extension, direction and magnitude of the retinal displacement as in the IR homography (A). c & d. IR images from postoperative months 1 (c) and 12 (d) with measurements of the distance between: Optic nerve landmarks (e & I blue circle), to retinal vessel bifurcation landmarks (f, j red circle and g, k green circle), and choroidal landmarks (h & l yellow circle). An increase in the distance to the retinal bifurcations at the 12-month follow-up is shown: Superior (f & j, red circle): 6947 μm vs. 7125 μm (Δ + 178), Inferior (g & k, green circle): 5835 μm vs. 6055 μm (Δ + 220). The distance to the choroidal landmarks remained approximately the same: Superior (h& l, yellow circle): 8153 μm vs. 8186 μm (Δ + 33), Inferior (c & d, orange circle): 7618 μm vs. 7617 μm (Δ −1)

*IR homography* is defined as the superimposition of two IR images using corresponding mapped points of the choroidal vasculature and optic nerve. Using custom software, an overlay of the two images was generated to assess retinal vessel alignment, as we described previously [12, 13]. At least 4 choroidal and two optic nerve head point correspondences between the separate images were manually marked. Two different pseudocolor channels, red and green, were used to correctly differentiate the two images and to facilitate the evaluation of their alignment. Additionally, for each set of marked images, the software generated a “flipping view” video which allowed the investigator to switch between the corresponding marked images to further assess proper image alignment. The “flipping view” was assessed and graded as “aligned” or “not aligned” by ER and KB independently. In cases where the flipping view was not satisfactory aligned, the point correspondences were manually revised until choroidal alignment was achieved. Unsuccessful alignment due to low-image quality resulted in exclusion. A multimodal imaging approach as an additional verification was applied in selected cases using a fundus photography homography composed of color fundus photography images from the same follow-up visits, (Fig. 1), and assessment of corresponding anatomical locations on cross-sectional SS-OCT.

## Grading of retinal displacement on IR homography

The presence of retinal vessel misalignment of a minimum ½ vessel diameter in magnitude was evaluated by two independent graders (ER and MP), and discordances were resolved by a third grader, (KB). The graders were masked to patient identification, surgery technique and detachment characteristics. Displacement direction of the proximal and distal major arcades segments was evaluated by three of the authors (VMT, ER, MP) and included superior/inferior/nasal and temporal directions with combinations.

### Statistical analysis

Inter- grader reliability for the presence of displacement was assessed using Cohen’s kappa. Associations between displacement of the retina, PVR grade, fovea-off duration and detachment extent in clock hours were evaluated using the Spearman’s rho, and the relationship between displacement and the use of PFCL and type of tamponade was investigated using the Phi Coefficient. Significance level was set at *p* < 0.05. All analyses were performed using IBM SPSS statistical software (version 26; IBM Corp, Armonk, New York, USA).

## Results

38 patients were included in the original study, with 2 lost to follow-up. Of the remaining 36 patients, 14 were excluded due to the development of ERM, 1 due to a macula-involving redetachment, 1 due to a macular hole, and 7 because of insufficient image quality to perform either a flipping view video (4) or IR homography (3), resulting in *n* = 13 patients with a median age of 62 years (IQR = 11) included in the analysis. Clinical characteristics and displacement directions are presented in Table 1.

**Table 1:**
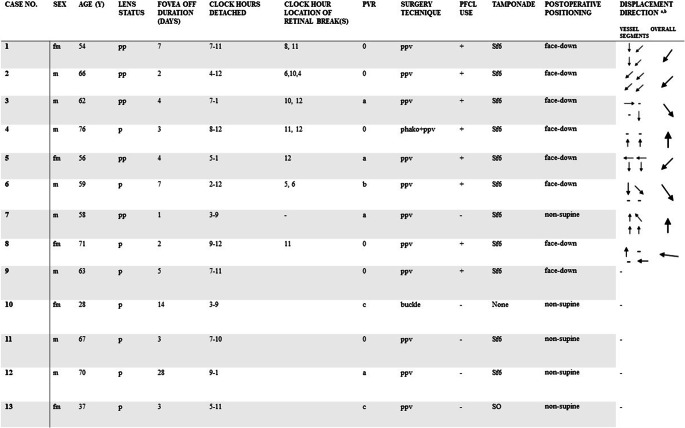
Clinical characteristics and displacement direction in eight patients with late retinal displacement

Vectors illustrate the displacement direction of the proximal and distal superior and inferior major vessel arcade segments and the overall direction

a: Left eyes have been right adjusted. b: Magnitude of vector does not correspond to magnitude of displacement fm, female; m, male; pp, pseudophakic; p, phakic; ppv, pars plana vitrectomy; phako, phacoemulsification; Sf6, sulphur hexafluoride; SO, silicon oil, PVR, proliferative vitreoretinopathy grade 0-C, PFCL, perfluocarbon liquid

The first IR images were captured at a median of 4 weeks after reattachment surgery (IQR = 11) and the second images at 24 months (IQR = 3).

8 patients (62%) had late displacement of the retina on IR homography. Inter-grader agreement was κ = 0.831 (SE: 0.16), (*p* = 0.002).

Displacement was positively associated with the use of PFCL, ϕ = 0.675, (*p* = 0.015), while no associations between displacement and detachment extent (r_s_=0.19, *p* = 0.52), fovea-off duration (r_s_ = −0.38, *p* = 0.19) PVR grade (r_s_ = −0.25, *p* = 0.41) or type of tamponade (ϕ = 0.56, *p* = 0.12) were found.

### Displacement characteristics of the reattached retina

**Fig. 2:**
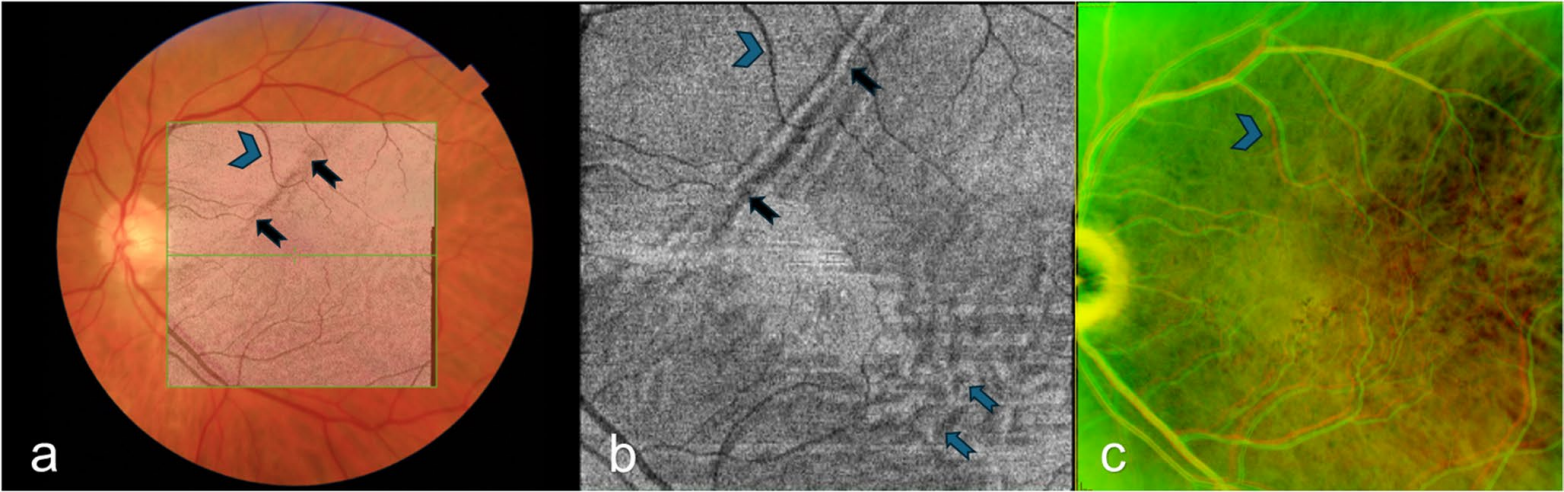
Late retinal displacement in previously attached macular area. Same patient as in Fig. 1. a + b: Postoperative month 1. a: Fundus photography with overlaying shadowgram (generated from the composite OCT cross-sectional scans) showing the margin of the previous retinal detachment, b: en face OCT segmented to the level of the ellipsoid zone and averaged to 15 μm to best represent the photoreceptors, showing ellipsoid zone disruption in areas previously detached as alternating hypo-and hyperreflectivity (blue arrows), c: IR homography composed from IR SLO images captured at the postoperative months 1- and 12, showing misalignment of the retinal vessels in previously attached macular area. Black arrows indicate the margin of the previous macular detachment extent. Blue arrowheads indicate corresponding retinal vessels. OCT: optical coherence tomography. SLO: scanning laser ophthalmoscopy

The overall direction of displacement occurred inferotemporally in three cases (38%), inferonasally in two (25%), superiorly in two (25%) and superotemporally in one case (13%). Two cases had retinal breaks both inferiorly and superiorly with a displacement direction inferiorly, four had superior breaks of which three had a displacement direction superiorly, and 1 case had inferior breaks with an inferiorly directed displacement (Table 1). In four cases, the magnitude of displacement was larger in the temporal segments of the vessel arcades than in the nasal. In three macula splitting detachment cases, retinal displacement was not confined to areas previously detached (Fig. 2).

## Discussion

This is the first study to present evidence for retinal displacement occurring after one month of reattachment to the RPE using a validated method. We found a late displacement of the reattached retina in 62% of patients and the most frequent direction of displacement was inferotemporal. Moreover, there was a tendency of the displacement direction to follow the location of the retinal break(s), and the magnitude of displacement increased with the distance from the optic nerve, possibly because of retinal tethering to the disc [15]. In three macula splitting detachment cases we found that displacement was not confined to previously detached macular areas, which may reflect a general tractional force on the retina.

Our findings are consisted with the observation by Shiragami et al. who found a change in the distance between the RVPs and the retinal vessels when comparing FAF images from the 3- and 12 months follow-up after RRD repair [2].

PFCL use and late displacement of the retina were positively associated. Although we cannot establish a causal relationship, this finding might indicate a potential long-term increased retinal plasticity induced by PFCL, warranting future investigation through multivariate analysis in a similar study with larger sample size.

Displacement of the retina with respect to its pre-detachment position visualized on FAF images, i.e. low integrity retinal attachment, is believed to be a result of SRF bulk flow during and immediately after reattachment surgery [4, 16], and is thought to be counteracted through immediate postoperative face-down positioning by limiting the effect of gravity [7, 9]. We could not establish a clear trend in the late displacement characteristics of the retina, but in over half of cases where the displacement occurred inferiorly, a continuous retinal stretch due to gravity may be an explanatory factor. Similarly, we hypothesize that the superiorly directed displacements are caused by a long-term susceptibility to tractional forces due to residual SRF and supine head positions, promoting fluid displacement in the direction of gravity. The temporally directed displacements may reflect increased tissue plasticity in the areas which were most frequently detached.

A long-term susceptibility to tractional forces may be a result of incomplete anatomical and metabolic recovery of the photoreceptor−RPE interface. Surviving photoreceptors start to regenerate the outer segments within hours after reattachment to the RPE, but a full recovery of the pre-detachment lengths takes up to several months [17–19]. Likewise, the RPE cells remodel their complex apical processes into a shortened and undifferentiated perimeter during a retinal detachment, they remain truncated one month after reattachment, and the time to full regeneration of the cone sheaths is likely even longer [19–21].

An additional potential contributor to our findings is neuroglia structural and molecular remodeling leading to a reduced inter-retinal layer or retina−RPE adhesion. In the healthy retina, Müller glia help maintain collagen crosslinking in the extracellular matrix (ECM) by synthesis, secretion and regulation of collagens and other ECM components [22, 23]. They also contribute to mechanical resilience and retinal stiffness by acting as “taut springs”, providing tensile strength [24, 25]. Müller cell hypertrophy, proliferation and altered protein expression is evident 3 days after experimental retinal detachment [26], and after 28 days of reattachment, structural and molecular changes are not fully reversed [27].

We acknowledge certain limitations to our study. The use of thirty-degree field of view IR images limited the evaluation of a displacement peripheral to the posterior pole, however, we believe that from a visual function perspective, macular displacement is more relevant to investigate. We cannot extrapolate when in the period between the 1 month and two-year follow-up the macular displacement occurred, and as we did not have information about a potential displacement from the pre-detachment position, we could not compare the characteristics of the two kinds of displacements.

Advancements in image overlay techniques, such as the use of en face OCT angiography images which can differentiate between the retinal layers involved in the displacement and the addition of Farneback optical flow displacement vectors may aid in elucidating the underlaying processes and the influencing forces on retinal displacement [28, 29]. Additional research avenues to be explored include whether retinal displacement in the late follow-up after RRD is associated with retinal, RPE and glial cell remodeling, or long-term visual function recovery.
